# Pharmacokinetic parameters explain the therapeutic activity of antimicrobial agents in a silkworm infection model

**DOI:** 10.1038/s41598-018-19867-0

**Published:** 2018-01-25

**Authors:** Atmika Paudel, Suresh Panthee, Makoto Urai, Hiroshi Hamamoto, Tomohiko Ohwada, Kazuhisa Sekimizu

**Affiliations:** 1 Institute of Medical Mycology, Teikyo University, Hachioji, Tokyo, Japan; 2 Department of Chemistry for Life Sciences and Agriculture, Faculty of Life Sciences, Tokyo University of Agriculture, Setagaya, Tokyo, Japan; 3 Laboratory of Organic and Medicinal Chemistry, Graduate School of Pharmaceutical Sciences, The University of Tokyo, Bunkyo, Tokyo, Japan; 4 Genome Pharmaceuticals Institute Co., Ltd., Bunkyo, Tokyo, Japan

## Abstract

Poor pharmacokinetic parameters are a major reason for the lack of therapeutic activity of some drug candidates. Determining the pharmacokinetic parameters of drug candidates at an early stage of development requires an inexpensive animal model with few associated ethical issues. In this study, we used the silkworm infection model to perform structure-activity relationship studies of an antimicrobial agent, GPI0039, a novel nitrofuran dichloro-benzyl ester, and successfully identified compound **5**, a nitrothiophene dichloro-benzyl ester, as a potent antimicrobial agent with superior therapeutic activity in the silkworm infection model. Further, we compared the pharmacokinetic parameters of compound **5** with a nitrothiophene benzyl ester lacking chlorine, compound **7**, that exerted similar antimicrobial activity but had less therapeutic activity in silkworms, and examined the metabolism of these antimicrobial agents in human liver fractions *in vitro*. Compound **5** had appropriate pharmacokinetic parameters, such as an adequate half-life, slow clearance, large area under the curve, low volume of distribution, and long mean residence time, compared with compound **7**, and was slowly metabolized by human liver fractions. These findings suggest that the therapeutic effectiveness of an antimicrobial agent in the silkworms reflects appropriate pharmacokinetic properties.

## Introduction

In most of the cases, antimicrobial agents that exhibit potent antimicrobial activity *in vitro* do not exhibit therapeutic activity in the animal infection models^1^. The lack of therapeutic activity of these compounds is due to their inappropriate pharmacokinetic properties, such as low bioavailability, short half-life, rapid metabolism, and rapid clearance resulting in a short duration of action that limits the drug from exerting the desired therapeutic effect^2^. For this reason, it is crucial to evaluate the pharmacokinetic properties of candidate compounds during the early phase of drug development. Several *in silico* and *in vitro* approaches are used to predict the pharmacokinetics of drug candidates^3–13^, but mimicking the complex environment of the animal models is difficult. *In vivo* tests in the mammalian models, however, are associated with the ethical issues and require the administration of a substantial amount of the compound, which is not always feasible at the early stage of drug development. An appropriate *in vivo* model is needed to evaluate the pharmacokinetic parameters following the administration of only a small quantity of the compound.

Silkworms (*Bombyx mori*) and mammals share some common features with regard to the absorption, distribution, and metabolism of xenobiotics^14^, and we previously demonstrated that clinically used antibiotics have therapeutic activities in infected silkworms with ED_50_ values similar to those in mammals^15^. Silkworms are economical, associated with fewer ethical issues, and share basic biological processes related to pharmacokinetics with mammals^16^. Previously, we utilized silkworms to identify therapeutically effective novel antimicrobial agents from microbial culture broths and chemical libraries: lysocin E^17,18^, ASP2397^19^, nosokomycin^17,20^, and GPI0363^21^, which have therapeutic effects in a mouse infection model. A detailed analysis of the pharmacokinetic parameters of antimicrobial agents in silkworms has not been performed, however, and could provide insight into the pharmacokinetics and therapeutic effectiveness of antimicrobial agents that could be developed for use in mammals.

In this research, we focused on the antimicrobial nitrofuran dichloro-benzyl ester GPI0039 (**1**), identified from chemical library screening^21^ with a weak therapeutic effectiveness in a silkworm infection model. We obtained more potent therapeutically effective antimicrobial agents and demonstrated a link between the pharmacokinetic properties and therapeutic effects in silkworms. Thus, drug seed selection based on therapeutic activity in a silkworm model may facilitate the discovery of drug-like compounds with the appropriate pharmacokinetic parameters.

## Results and Discussion

### Screening and identification of therapeutically effective antimicrobial agents

**Figure 1:**
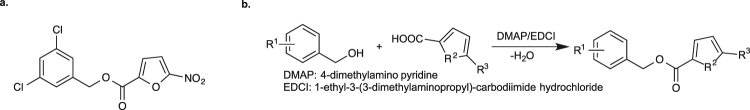
GPI0039 (**1**) chemical structure and synthesis scheme. (**a**) Chemical structure of **1** and (**b**) Scheme for the chemical synthesis of **1** and other derivatives.

Drug-resistant *Staphylococcus aureus* is a global problem^22–28^ and viewed as a serious public health threat^29,30^. To select antimicrobial agents effective against drug-resistant *S*. *aureus*, we used methicillin-resistant *S*. *aureus* (MRSA) for *in vitro* screening. We screened a chemical library of the Drug Discovery Initiative at the University of Tokyo for compounds that inhibit the growth of MRSA4 *in vitro*, and among 103,873 compounds, we obtained 3383 compounds that inhibited the growth of MRSA4^21^. One of these was GPI0039 (**1**) (Fig. 1a), a compound that displayed antimicrobial activity against MRSA4 with a minimum inhibitory concentration (MIC) of 12.5 μg/ml. GPI0039 also slightly prolonged the survival of the *S*. *aureus*-infected silkworms. Given that **1** is a nitrofuran benzyl ester, we thought that both the substituents, benzyl and furan groups, could be modified for extensive structure-activity relationship studies aiming to improve the therapeutic activity of **1** in the silkworm.

**Figure 2:**
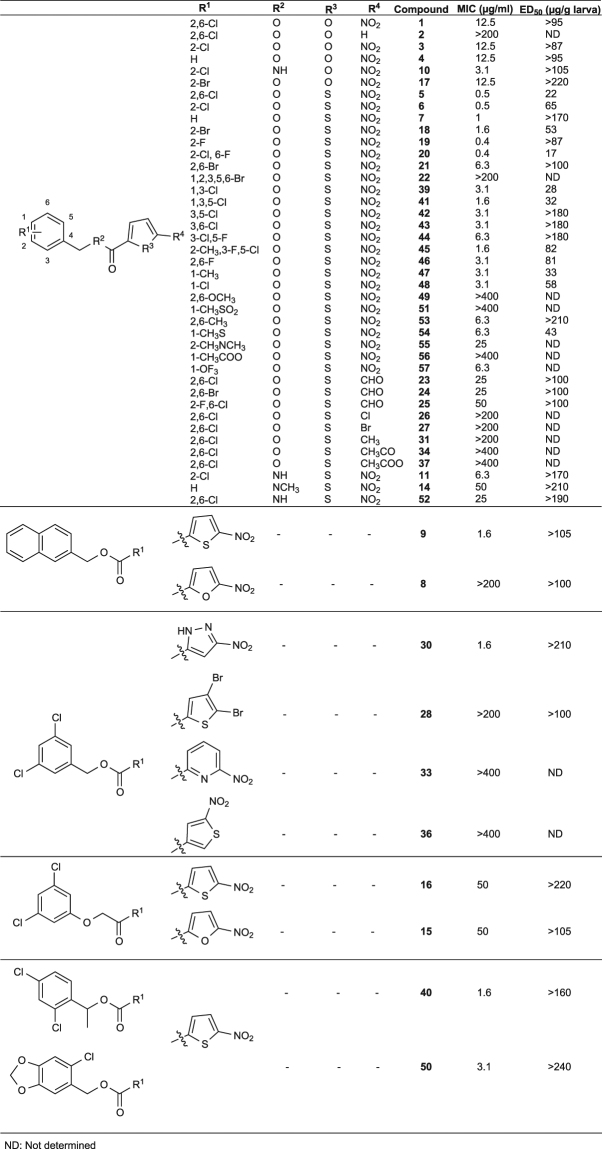
Structure activity relationship of GPI0039. Derivatives of GPI0039 were synthesized and *in vitro* antimicrobial activities as well as therapeutic activities in the silkworm infection model were determined.

We synthesized 50 compounds (Fig. 2, Supplementary Table 1**)** using the Steglich esterification scheme^31^ in the presence of a coupling agent, 4-dimethylamino pyridine, and a catalyst, 1-ethyl-3-(3-dimethylaminopropyl)-carbodiimide hydrochloride **(**Fig. 1b**)**. Antimicrobial activities of the compounds were examined by determining the MIC against *S*. *aureus* MRSA4 *in vitro*; therapeutic activities were examined using a systemic infection model of silkworms infected with *S*. *aureus* MSSA1. In comparison with **1**, 25 compounds exhibited enhanced antimicrobial activity (MIC < 12.5 µg/ml) and 11 of these displayed superior therapeutic effects in *S*. *aureus*-infected silkworms based on the ED_50_ values (Fig. 2).

Substitution of the nitrofuran ring in **1**, **3**, **4**, **8**, and **17** with a nitrothiophene ring in **5**, **6**, **7**, **9**, and **18**, respectively, enhanced the antimicrobial activities of all the derivatives, and increased the therapeutic effectiveness in **5**, **6**, and **18**, suggesting the need for the nitrothiophene moiety. In compounds with a nitrothiophene ring, substitution of halogen in the benzene ring did not affect the antimicrobial activities, except **22**, but markedly affected the therapeutic activities; some compounds had increased therapeutic activity (**5**, **6**,**18**, **20**, **39**, **41**, **45**, **46**, **48**), while others had decreased therapeutic activity (**7**, **19**, **21**, **42**, **43**, **44**). A closer look at the structures suggested the importance of the 2,6 dichloro-benzyl moiety for the therapeutic activity. Other modifications in the thiophene ring (**23**, **24**, **25**, **26**, **27**, **28**, **31**, **34**, **37**) or benzene ring (**47**, **49**, **51**, **53**, **54**, **55**, **56**, **57**) or both rings (**8**, **9**, **30**, **28**, **33**, **36**, **50**, **15**, **16**, **40**) did not remarkably affect the activities. Taken together, these findings suggested that both nitrothiophene and 2,6-dichloro-benzene are important for the therapeutic activity in the silkworm infection model.

### Antimicrobial spectrum of compounds 5 and 7

Based on the structure-activity analysis, we found several compounds, e.g., **5**, **6**, **7**, **39**, **41**, **42**, **43** and **48**, had similar structures but better antimicrobial activity than the original hit **1**, but the therapeutic activity in the silkworm infection model differed markedly among compounds **(**Fig. 2**)**. For example, compound **5** had superior therapeutic activity in *S*. *aureus*-infected silkworms (ED_50_: 22 µg/g) compared with compound **7** (ED_50_: > 170 µg/g), although their MIC values against *S*. *aureus* MSSA1 were similar [0.5 µg/ml (**5**) and 1 µg/ml (**7**)]. Interestingly, these two compounds only differed in the presence of chlorine in the benzene ring; compound **5** is a nitrothiophene dichloro-benzyl ester, while compound **7** is a nitrothiophene benzyl ester lacking chlorine. An extended antimicrobial spectrum revealed that both compounds had similar antimicrobial activity against a wide range of Gram-positive bacteria (Table 1). Despite their similar antimicrobial spectrums, compound **5** had superior therapeutic activity compared with compound **7**. Thus, based on the differences in their therapeutic activities in the silkworm infection model, we could select the compound with higher therapeutic activity.

**Table 1:** Antimicrobial spectrum of compounds **5** and **7**. Minimum inhibitory concentration (MIC) was determined against bacteria by broth microdilution assay. Data represent median of three experiments.

Bacteria	MIC (µg/ml)	
	5	7
Methicillin-susceptible *Staphylococcus aureus* (MSSA)		
MSSA1 (clinical isolate)	0.5	1
Newman	0.5	1
Smith ATCC13709	0.5	0.5
RN4220	0.5	1
NCTC8325	0.5	1
Methicillin-resistant *S*. *aureus* (MRSA)		
MRSA4 (clinical isolate)	0.5	1
USA300 FPR3757 (clinical isolate)	1	1
*Staphylococcus pseudintermedius* JCM17571	0.125	0.5
*Staphylococcus haemolyticus* JCM2416	0.25	0.25
*Staphylococcus simulans*	0.25	0.5
*Bacillus subtilis* JCM2499	1	1
*Bacillus cereus* JCM20037	1	1
*Streptococcus pneumoniae* (clinical isolate)	1	1
*Streptococcus pyogenes* SSI-9	8	16
*Streptococcus sanguinis* JCM5708	>128	>128
*Streptococcus agalactiae* JCM5671	>128	>128
*Enterococcus faecalis* EF1	>128	>128
Vancomycin- resistant *E*. *faecalis* EF5	>128	>128
*Listeria monocytogenes* 10403S	>128	>128
*Escherichia coli* W3110	>128	>128
*Pseudomonas aeruginosa* PAO1	>128	>128

### Evaluation of the pharmacokinetic parameters of compounds 5 and 7

**Figure 3:**
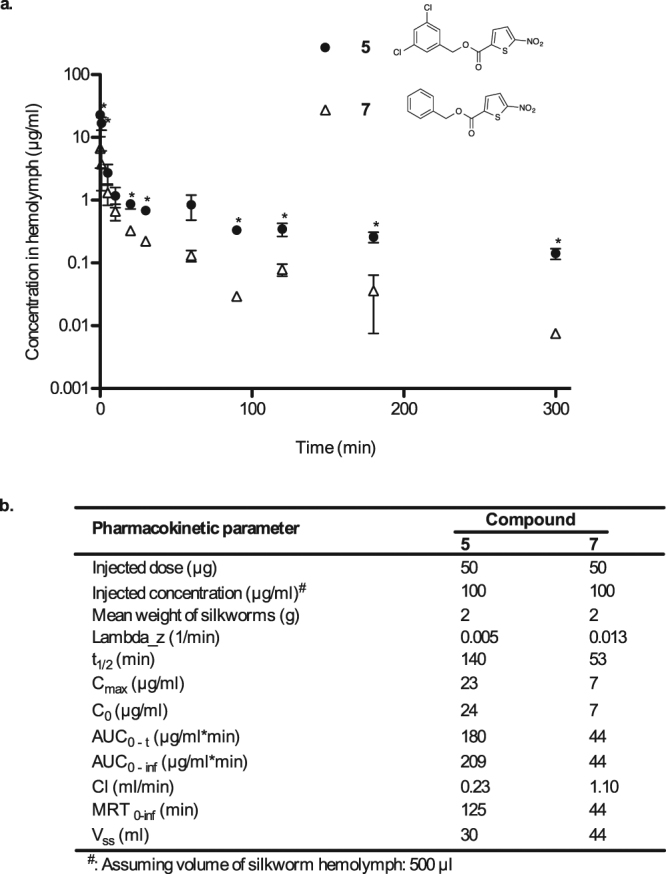
Exponential decay of the compounds with time after intra-hemolymph administration. (**a**) Chemical structures and the concentrations of compound **5** (•) and **7** (∆) in the silkworm hemolymph. The results are expressed as mean ± SEM of triplicate experiments (*indicates p ≤ 0.05 as analyzed by Student’s *t*-test). (**b**) Pharmacokinetic parameters of compounds **5** and **7** in silkworm hemolymph obtained using the PKSolver add-in for Excel. (C_0_: maximum plasma concentrations based on the extrapolated time-zero value; AUC_0-t_: area under the concentration-time curve from 0 to the last measured value; AUC_0-inf_: extrapolated area under the concentration-time curve from 0 to infinity; t_1/2_: half-life at terminal phase; Cl: total clearance; V_ss_: volume of distribution at steady state; MRT: mean residence time).

To gain an insight into the reason for the difference in the therapeutic activities of compounds **5** and **7**, we evaluated the pharmacokinetic properties of both compounds *in vivo* in silkworms. We injected silkworms into the hemolymph with compound **5** or compound **7** (50 µg each) and collected the hemolymph from each injected silkworm at different time-points for high performance liquid chromatography (HPLC) analysis. The concentration-time profile was plotted and the pharmacokinetic parameters were analyzed using PKSolver^32^ (Fig. 3a,b). The initial concentrations (C_0_) of compounds **5** and **7** were 24 and 7 µg/ml, respectively, indicating that compound **7** had a higher distribution capacity than compound **5**. The volume of distribution at steady state (V_ss_) value of compound **7** was larger than that of compound **5**, further indicating the higher distribution capacity of compound **7** compared with compound **5**. The concentrations of compounds **5** and **7** in the hemolymph decreased with time, with compound **7** having faster rate of elimination than compound **5**. At 5 h, the concentration of compound **5** in the hemolymph was approximately 10-fold higher than that of **7**. The half-life of compounds **5** and **7** were 140 min and 53 min, with a mean residence time of 125 min and 44 min, respectively. Both the area under the curves (AUC) at 5 h and that extrapolated to infinity were higher for compound **5** than for compound **7** in the silkworm.

**Figure 4:**
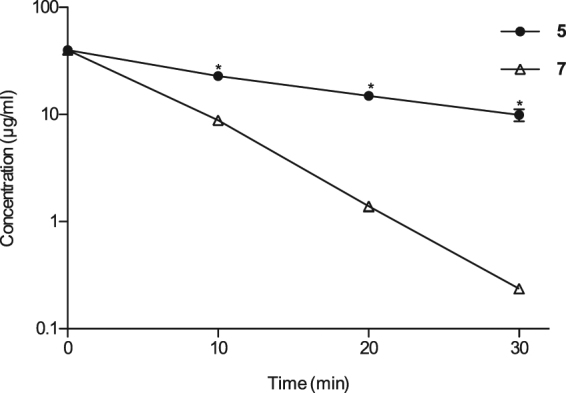
Concentration of compounds **5** and **7** after treatment with human liver S9 fractions over time. Compound **5** and **7** (40 µg/ml) were treated with human liver S9 fractions, then samples were aliquoted at the indicated time, analyzed by HPLC, and concentration at each time-point was determined. Data are shown as mean ± SEM of triplicate experiments (*indicates p ≤ 0.05 as analyzed by Student’s *t*-test).

The pharmacokinetic analysis provided a plausible explanation for the lower ED_50_ of compound **5** compared with compound **7** in the silkworm infection model. Additionally, the longer half-life of compound **5** suggested its slower metabolism. Therefore, to evaluate the metabolic stability of these compounds in mammals, we performed an *in vitro* assay using pooled human liver S9 fractions. Compound **5** was slowly metabolized compared with compound **7** (Fig. 4). These findings together with the longer mean residence time, higher AUC, and slower clearance of compound **5** further explain its higher therapeutic activity in the silkworm infection model, and reveal a correlation between therapeutic activity and the pharmacokinetic properties. In addition, our findings suggested that compounds with higher stability in silkworms are also slowly metabolized in mammals.

Clinically used antibiotics have the desired pharmacokinetics, which has also been demonstrated in the *Galleria mellonella* infection model^33,34^. In the present study, we demonstrated a correlation between the pharmacokinetic parameters and therapeutic activity for a new antimicrobial agent using the silkworm infection model. A major criterion for the development of antimicrobial agents as drugs is therapeutic activity, and pharmacokinetic parameters reflect the duration of action of a drug in the host; antimicrobial agents exhibiting the appropriate pharmacokinetic parameters are more likely to exert therapeutic activity. Based on the therapeutic activity of novel therapeutically effective antimicrobial compounds discovered using the silkworm infection model^17,19–21^ in a mouse systemic infection model^17,19,21^, and the correlation of pharmacokinetic parameters with therapeutic activity, silkworms are useful animals for selecting therapeutically effective compounds with the appropriate pharmacokinetic properties.

## Conclusion

The findings of the present study revealed that antimicrobial agents exhibiting the appropriate pharmacokinetic properties *in vitro* and *in vivo* also exhibit therapeutic activities in a silkworm infection model. By synthesizing derivatives of a lead compound guided by therapeutic effectiveness in a silkworm infection model, more potent therapeutically effective compounds can be identified. Our results demonstrate that therapeutic effectiveness is governed by pharmacokinetics in silkworms as in mammals. The higher stability of compound **5** in silkworms and lower metabolism by human liver fraction, and the correlation between the therapeutic activity and pharmacokinetic properties in silkworms demonstrate the usefulness of silkworms for the initial screening and pharmacokinetic evaluation of antimicrobial agents.

## Materials and Methods

### Chemical library screening

A total of 108,373 compounds from the chemical library of the Drug Discovery Initiative at the University of Tokyo were screened for antibacterial activity against *S*. *aureus* strain MRSA4 as previously reported^21^. Those compounds that inhibited the growth of MRSA4 at 100 µM were further evaluated for their therapeutic effects in silkworms infected with *S*. *aureus* strain MSSA1 according to previously reported studies^17,21^.

### Synthesis of derivatives

Reagents for the synthesis of the derivatives were purchased from Wako Pure Chemicals (Tokyo, Japan). Derivatives were synthesized by Steglich esterification scheme^31^ in the presence of a coupling agent 4-dimethylamino pyridine (Wako Pure Chemicals) and a catalyst, 1-ethyl-3-(3-dimethylaminopropyl)-carbodiimide hydrochloride (Tokyo Chemical Industry Co. Ltd., Tokyo, Japan).

### Antimicrobial spectrum analysis

Antimicrobial activity was assessed by determining the MIC by broth dilution assay according to our previous reports^21,35,36^ as per the Clinical and Laboratory Standards Institute^37^. Briefly, serial dilutions of compound **5**, compound **7**, and positive controls were prepared in cation-adjusted Mueller-Hinton Broth (MHB, Difco, Franklin Lakes, NJ, USA), 100 µl of which was dispensed into each well of a round-bottomed 96-well plate. The final concentrations in the well ranged from 128 µg/ml to 0.0625 µg/ml. Bacteria were grown at 37 °C overnight on either Luria Bertani (tryptone 10 g/l, yeast extract 5 g/l, NaCl 10 g/l) or Tryptic Soy Broth (TSB, Difco) agar plates or sheep blood agar plates (Eiken Chemical Co. Ltd., Tokyo, Japan). Bacterial inoculum was prepared by direct suspension of the colonies in 0.9% saline, and adjusted to obtain a turbidity equivalent to a 0.5 McFarland standard using a spectrophotometer UV-1280 (Shimadzu Corp., Kyoto, Japan). The suspension was then diluted 1:20 in cation-adjusted MHB (Difco), and 10 µl of it was inoculated into each well of previously prepared 96-well plates containing compounds to obtain approximately 5 × 10^4^ colony forming units/well and incubated at 37 °C for 20 h. For Streptococcus spp, cation-adjusted MHB with 2.5% lysed horse blood (Nippon Biotest Laboratories Inc, Tokyo, Japan), pH 7.4 was used and the MIC was determined at 24 h. Vancomycin (Wako Pure Chemicals) or norfloxacin (Sigma Aldrich, St Louis, MO, USA) were used as positive controls. Each plate contained a growth control well and a vehicle control well. The MIC was determined as the minimum concentration that inhibited the growth of the bacteria.

### Pharmacokinetic parameters analysis

Silkworms larvae hatched from eggs (Hu•Yo × Tsukuba•Ne, Ehime Sanshu, Ehime, Japan) were fed Silkmate 2S (Nosan Corporation, Yokohama, Japan) and grown at 27 °C until the fourth molt stage. They were, then, fed an antibiotic-free artificial food, Silkmate (Katakura Industries Co., Ltd., Tokyo, Japan), on the first day of fifth-instar larval stage. The next day, larvae (~2 g) were injected into the hemolymph with 50 µg of compounds **5** and **7** using a 1 ml syringe with a 27 G needle (Terumo Corporation, Tokyo, Japan). Silkworms were incubated after injection at 27 °C without feeding. The hemolymph was harvested from the limbs of individual silkworms at the indicated time, and the silkworms were killed after the harvest. The harvested hemolymph was mixed with an equal volume of acetonitrile followed by centrifugation at 15, 000 rpm for 10 min at 4 °C. The supernatant was collected, evaporated, and dissolved into 50% acetonitrile. The samples thus prepared were analyzed by high performance liquid chromatography (HPLC) equipped with a Waters 2998 Photodiode Array Detector (Waters Corporation, Milford, MA, USA) and a Waters 2707 Autosampler (Waters) using a Senshu-Pak Pegasil ODS-SP-100 column (4.6 Ø × 250 mm; Senshu Scientific, Tokyo, Japan) with gradient elution of 50–100% acetonitrile for 30 min at a flow rate of 1 ml/min. Standard curves for each compound were drawn by measuring the peak area on HPLC charts, and the amounts of each compound in the sample were determined. Mean concentrations from triplicate experiments were determined for each time-point and the pharmacokinetic parameters were determined using the PKSolver 2.0 ‘add-in’ for Excel 2007 on a computer running Windows 7^32^. Non-compartment analysis was performed with area under the curve calculated using the linear trapezoidal method. The terminal elimination slope, λ_z_ was calculated based on default parameters. Statistical analysis was performed using Prism 5 for Mac OS X, version 5.0 f (GraphPad Software, La Jolla, CA).

### Metabolism of compounds in human liver S9 fractions

Metabolism of the compounds was determined according to Yang *et al*.^38^ with slight modifications. Briefly, reaction mixture containing 5 mM MgCl_2_, 2 mM NADPH, 20 µg/ml pooled human liver S9 fractions (Sekisui Xenotech, KS, USA) in 0.05 M Tris-HCl buffer (pH 7.4) was pre-incubated at 37 °C for 5 min. The reaction was initiated by the addition of 40 µg/ml compound **5** or compound **7** and incubation was continued. Aliquots (100 µl) were collected at 10, 20, and 30 min, and an equal volume of acetonitrile was added. The resulting mixture was analyzed by HPLC equipped with a Waters 2998 Photodiode Array Detector and a Waters 2707 Autosampler using a Senshu-Pak Pegasil ODS-SP-100 column (4.6 Ø × 250 mm) with gradient elution of 50–100% acetonitrile for 30 min at the flow rate of 1 ml/min. Statistical analysis was performed using Prism 5 for Mac OS X, version 5.0 f (GraphPad Software).

## Electronic supplementary material

Supplementary Table
