# Supplementary material for: Pharmacokinetic parameters explain the therapeutic activity of antimicrobial agents in a silkworm infection model
Source: Sci Rep. 2018 Jan 25;8:1578. doi: 10.1038/s41598-018-19867-0 (PMC5785531; doi:10.1038/s41598-018-19867-0)
Supplement: Supplementary file 1 — Supplementary Table [file 41598_2018_19867_MOESM1_ESM.pdf]

Supplementary information:

Pharmacokinetic parameters explain the therapeutic activity of antimicrobial agents in a silkworm infection model

Atmika Paudel[1], Suresh Panthee[1], Makoto Urai[2], Hiroshi Hamamoto[1], Tomohiko Ohwada[3]*, Kazuhisa Sekimizu[1,4]*

[1]Institute of Medical Mycology, Teikyo University, Hachioji, Tokyo, Japan.

[2]Department of Chemistry for Life Sciences and Agriculture, Faculty of Life Sciences, Tokyo University of Agriculture, Setagaya, Tokyo, Japan.

[3]Laboratory of Organic and Medicinal Chemistry, Graduate School of Pharmaceutical Sciences, The University of Tokyo, Bunkyo, Tokyo, Japan.

[4]Genome Pharmaceuticals Institute Co., Ltd., Bunkyo, Tokyo, Japan.

*Corresponding authors:

Prof. Kazuhisa Sekimizu
Teikyo University Institute of Medical Mycology, 359 Otsuka, Hachioji, Tokyo 192-0395, Japan.
sekimizu@main.teikyo-u.ac.jp

Prof. Tomohiko Ohwada
Laboratory of Organic and Medicinal Chemistry, Graduate School of Pharmaceutical Sciences, The University of Tokyo, Bunkyo, Tokyo 113-0033, Japan.
ohwada@mol.f.u-tokyo.ac.jp

Supplementary Table 1

Compound	Structure	NMR analysis
**1**	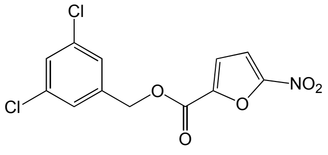	^{1}H NMR (500 MHz, $CDCl_3$) δ 7.37 ppm (dd, *J* = 2.2, 1.7 Hz, 1H), 7.36 (d, *J* = 3.4 Hz, 1H), 7.34 (d, *J* = 3.9 Hz, 1H), 7.32 (d, *J* = 1.7 Hz, 2H), 5.33 (s, 2H); ^{13}C NMR (126 MHz, $CDCl_3$) δ ppm 156.53, 152.64, 144.16, 137.79, 135.42, 129.03, 126.86, 119.54, 111.46, 66.04. Anal. calcd for $C_{12}H_7Cl_2NO_5$: C, 45.60; H, 2.23; N, 4.43; Found: C, 45.56; H, 2.34; N, 4.41.
**2**	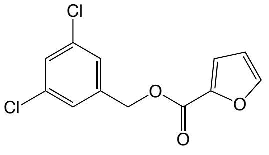	^{1}H NMR (500 MHz, $CDCl_3$) δ 7.61 ppm (d, *J* = 1.7 Hz, 1H), 7.33 (dd, *J* = 1.7, 1.7 Hz, 1H), 7.31 (d, *J* = 2.2 Hz, 2H), 7.25 (d, *J* = 3.4 Hz, 1H), 6.54 (dd, *J* = 3.4, 1.7 Hz, 1H), 5.27 (s, 2H); ^{13}C NMR (126 MHz, $CDCl_3$) δ ppm 158.13, 146.81, 144.06, 138.93, 135.25, 128.54, 126.50, 118.77, 112.03, 64.81. Anal. calcd for $C_{12}H_8Cl_2O_3$: C, 53.17; H, 2.97; Found: C, 52.88; H, 3.01.
**3**	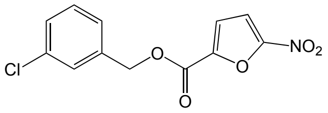	^{1}H NMR (500 MHz, $CDCl_3$) δ 7.44 ppm (bs, 1H), 7.35 – 7.32 (m, 5H), 5.36 (s, 2H); ^{13}C NMR (126 MHz, $CDCl_3$) δ ppm 156.68, 152.59, 144.44, 136.48, 134.63, 130.09, 129.04, 128.67, 126.70, 119.31, 111.51, 66.86. Anal. calcd for $C_{12}H_8ClNO_5$: C, 51.17; H, 2.86; N, 4.97; Found: C, 51.02; H, 2.95; N, 4.90.
**4**	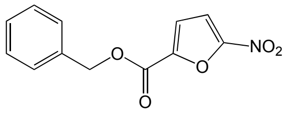	^{1}H NMR (500 MHz, $CDCl_3$) δ 7.48 – 7.36 ppm (m, 5H), 7.33 (d, *J* = 4.0 Hz, 1H), 7.30 (d, *J* = 4.0 Hz, 1H), 5.40 (s, 2H); ^{13}C NMR (126 MHz, $CDCl_3$) δ ppm 156.85, 152.47, 144.75, 134.57, 128.89, 128.77, 128.73, 119.06, 111.51, 67.87.
**5**	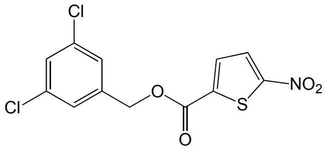	^{1}H NMR (500 MHz, $CDCl_3$) δ 7.89 ppm (d, *J* = 4.5 Hz, 1H), 7.74 (d, *J* = 4.0 Hz, 1H), 7.38 (dd, *J* = 2.3, 1.7 Hz, 1H), 7.31 (d, *J* = 1.7 Hz, 2H), 5.31 (s, 2H); ^{13}C NMR (126 MHz, $CDCl_3$) δ ppm 160.19, 155.86, 137.95, 137.60, 135.45, 132.25, 128.98, 127.94, 126.65, 66.17. Anal. calcd for $C_{12}H_7Cl_2NO_4S$: C, 43.39; H, 2.12; N, 4.22; Found: C, 43.21; H, 2.31; N, 4.07.
**6**	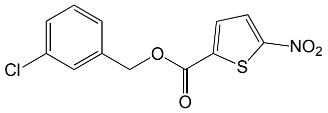	^{1}H NMR (500 MHz, $CDCl_3$) δ 7.87 ppm (d, *J* = 3.9 Hz, 1H), 7.73 (d, *J* = 3.9 Hz, 1H), 7.42 (bs, 1H), 7.37 – 7.34 (m, 2H), 7.33 – 7.29 (m, 1H), 5.34 (s, 2H); ^{13}C NMR (126 MHz, $CDCl_3$) δ ppm 160.31, 155.67, 138.00, 136.66, 134.67, 132.04, 130.08, 128.96, 128.42, 127.94, 126.43, 66.96. Anal. calcd for $C_{12}H_8ClNO_4S$: C, 48.41; H, 2.71; N, 4.70; Found: C, 48.27; H, 2.76; N, 4.68.
**7**	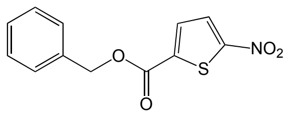	^{1}H NMR (500 MHz, $CDCl_3$) δ 7.86 ppm (d, *J* = 4.5 Hz, 1H), 7.71 (d, *J* = 4.5 Hz, 1H), 7.45 – 7.35 (m, 5H), 5.37 (s, 2H); ^{13}C NMR (126 MHz, $CDCl_3$) δ ppm 160.46, 155.51, 138.43, 134.73, 131.82, 128.79, 128.76, 128.43, 127.92, 67.95.
**8**	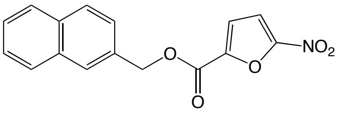	^{1}H NMR (500 MHz, $CDCl_3$) δ 7.92 ppm (bs, 1H), 7.90 – 7.84 (m, 3H), 7.56 – 7.50 (m, 3H), 7.33 (d, *J* = 3.4 Hz, 1H), 7.31 (d, *J* = 3.4 Hz, 1H), 5.56 (s, 2H); ^{13}C NMR (126 MHz, $CDCl_3$) δ ppm 156.86, 152.54, 144.75, 133.33, 133.11, 131.94, 128.65, 128.20, 128.06, 127.73, 126.65, 126.51, 125.99, 119.05, 111.44, 68.03.
**9**	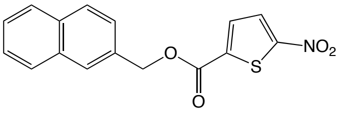	^{1}H NMR (500 MHz, $CDCl_3$) δ 7.91 – 7.84 ppm (m, 5H), 7.73 (d, *J* = 4.5 Hz, 1H), 7.54 – 7.50 (m, 3H), 5.54 (s, 2H); ^{13}C NMR (126 MHz, $CDCl_3$) δ ppm 160.50, 155.53, 138.42, 133.30, 133.11, 132.09, 131.86, 128.68, 128.04, 127.93, 127.75, 126.63, 126.53, 125.81, 68.15. Anal. calcd for $C_{16}H_{11}NO_4S$: C, 61.33; H, 3.54; N, 4.47; Found: C, 61.09; H, 3.67; N, 4.42.
**10**	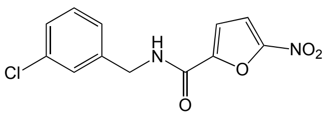	^{1}H NMR (500 MHz, $CDCl_3$) δ 7.38 ppm (d, *J* = 3.9 Hz, 1H), 7.34 (bs, 1H), 7.32 – 7.29 (m, 3H), 7.25 – 7.22 (m, 1H), 6.91 (s, 1H), 4.62 (d, *J* = 6.2 Hz, 2H); ^{13}C NMR (126 MHz, $CDCl_3$) δ ppm 156.26, 151.28, 147.71, 139.08, 134.75, 130.17, 128.17, 128.06, 126.09, 116.37, 112.45, 42.96. Anal. calcd for $C_{12}H_9ClN_2O_4$: C, 51.35; H, 3.23; N, 9.98; Found: C, 51.32; H, 3.37; N, 9.97.

Compound	Structure	NMR analysis
**11**	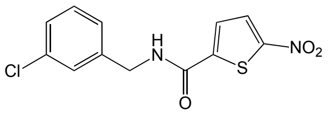	^{1}H NMR (500 MHz, $CDCl_3$) δ 7.86 ppm (d, *J* = 4.0 Hz, 1H), 7.38 (d, *J* = 4.0 Hz, 1H), 7.32 (bs, 1H), 7.31 – 7.29 (m, 2H), 7.25 – 7.21 (m, 1H), 6.48 (s, 1H), 4.61 (d, *J* = 5.7 Hz, 2H); ^{13}C NMR (126 MHz, $CDCl_3$) δ ppm 159.94, 154.40, 144.13, 139.04, 134.80, 130.26, 128.26, 128.13, 128.06, 126.15, 126.13, 43.78. Anal. calcd for $C_{12}H_9ClN_2O_3S$: C, 48.57; H, 3.06; N, 9.44; Found: C, 48.45; H, 3.14; N, 9.40.
**14**	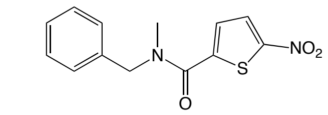	^{1}H NMR (500 MHz, $CDCl_3$) δ 7.78 ppm (bs, 1H), 7.45 – 7.18 (m, 6H), 4.75 (s, 2H), 3.13 (bs, 3H); ^{13}C NMR (126 MHz, $CDCl_3$) δ ppm 163.20, 153.50, 143.97, 135.74, 129.07, 128.02, 127.49, 53.42, 35.66. Anal. calcd for $C_{13}H_{12}N_2O_3S$: C, 56.51; H, 4.38; N, 10.14; Found: C, 56.43; H, 4.36; N, 10.11.
**15**	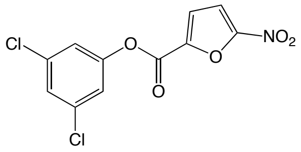	^{1}H NMR (500 MHz, $CDCl_3$) δ 7.50 ppm (d, *J* = 3.4 Hz, 1H), 7.43 (d, *J* = 3.4 Hz, 1H), 7.34 (dd, *J* = 1.7, 1.7 Hz, 1H), 7.21 (d, *J* = 1.7 Hz, 2H); ^{13}C NMR (126 MHz, $CDCl_3$) δ ppm 154.42, 152.97, 150.14, 143.21, 135.62, 127.22, 120.83, 120.50, 111.49. Anal. calcd for $C_{11}H_5Cl_2NO_5$: C, 43.74; H, 1.67; N, 4.64; Found: C, 43.71; H, 1.90; N, 4.54.
**16**	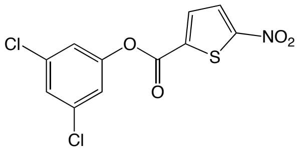	^{1}H NMR (500 MHz, $CDCl_3$) δ 7.94 ppm (d, *J* = 4.5 Hz, 1H), 7.88 (d, *J* = 4.5 Hz, 1H), 7.33 (dd, *J* = 1.7, 1.7 Hz, 1H), 7.21 (d, *J* = 1.7 Hz, 2H); ^{13}C NMR (126 MHz, $CDCl_3$) δ ppm 158.34, 156.59, 150.48, 136.37, 135.61, 133.45, 128.04, 127.17, 120.58. Anal. calcd for $C_{11}H_5Cl_2NO_4S$: C, 41.53; H, 1.58; N, 4.40; Found: C, 41.47; H, 1.73; N, 4.36.
**17**	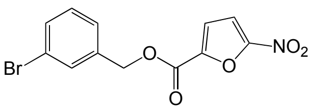	^{1}H NMR (500 MHz, $CDCl_3$) δ 7.59 ppm (bs, 1H), 7.51 (bd, *J* = 7.9 Hz, 1H), 7.38 (bd, *J* = 7.9 Hz, 1H), 7.34 (d, *J* = 3.4 Hz, 1H), 7.32 (d, *J* = 3.9 Hz, 1H), 7.27 (dd, *J* = 7.9, 7.9 Hz, 1H), 5.36 (s, 2H); ^{13}C NMR (126 MHz, $CDCl_3$) δ ppm 156.67, 152.62, 144.46, 136.79, 131.99, 131.59, 130.36, 127.19, 122.75, 119.31, 111.48, 66.79. Anal. calcd for $C_{12}H_8BrNO_5$: C, 44.20; H, 2.47; N, 4.30; Found: C, 44.12; H, 2.66; N, 4.28.
**18**	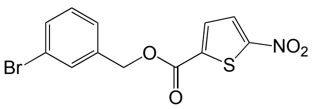	^{1}H NMR (500 MHz, $CDCl_3$) δ 7.87 ppm (d, *J* = 4.5 Hz, 1H), 7.73 (d, *J* = 3.9 Hz, 1H), 7.58 (bs, 1H), 7.51 (bd, *J* = 8.4 Hz, 1H), 7.36 (bd, *J* = 7.9 Hz, 1H), 7.28 (dd, *J* = 7.9, 7.9 Hz, 1H), 5.33 (s, 2H); ^{13}C NMR (126 MHz, $CDCl_3$) δ ppm 160.34, 155.70, 138.03, 136.95, 132.08, 131.93, 131.40, 130.38, 127.97, 126.97, 122.79, 66.93. Anal. calcd for $C_{12}H_8BrNO_4S$: C, 42.12; H, 2.36; N, 4.09; Found: C, 42.09; H, 2.47; N, 4.00.
**19**	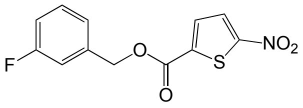	^{1}H NMR (500 MHz, $CDCl_3$) δ 7.87 ppm (d, *J* = 4.5 Hz, 1H), 7.73 (d, *J* = 4.5 Hz, 1H), 7.38 (ddd, *J* = 7.9, 7.9, 5.6 Hz, 1H), 7.20 (bd, *J* = 7.3 Hz, 1H), 7.13 (ddd, *J* = 9.5, 2.0, 2.0 Hz, 1H), 7.07 (ddd, *J* = 8.4, 8.4, 2.2 Hz, 1H), 5.36 (s, 2H); ^{13}C NMR (126 MHz, $CDCl_3$) δ ppm 162.88, 160.35, 155.68, 138.07, 137.13, 132.05, 130.43, 127.97, 123.82, 115.76, 115.22, 67.01. Anal. calcd for $C_{12}H_8FNO_4S$: C, 51.24; H, 2.87; N, 4.98; Found: C, 51.16; H, 2.98; N, 4.95.
**20**	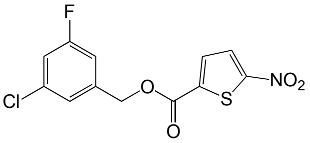	^{1}H NMR (500 MHz, $CDCl_3$) δ 7.89 ppm (d, *J* = 4.5 Hz, 1H), 7.75 (d, *J* = 4.5 Hz, 1H), 7.21 (bs, 1H), 7.11 (ddd, *J* = 7.9, 2.0, 2.0 Hz, 1H), 7.05 (ddd, *J* = 9.1, 1.8, 1.8 Hz, 1H), 5.32 (s, 2H); ^{13}C NMR (126 MHz, $CDCl_3$) δ ppm 162.7, 155.8, 160.20, 138.29, 137.62, 135.57, 132.24, 127.96, 124.09, 116.60, 113.62, 66.22. Anal. calcd for $C_{12}H_7ClFNO_4S$: C, 45.65; H, 2.23; N, 4.44; Found: C, 45.67; H, 2.38; N, 4.37.
**21**	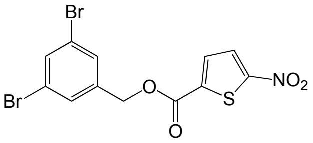	^{1}H NMR (500 MHz, $CDCl_3$) δ 7.88 ppm (d, *J* = 3.9 Hz, 1H), 7.74 (d, *J* = 3.9 Hz, 1H), 7.68 (dd, *J* = 1.7, 1.7 Hz, 1H), 7.51 (d, *J* = 1.7 Hz, 2H), 5.29 (s, 2H); ^{13}C NMR (126 MHz, $CDCl_3$) δ ppm 160.21, 155.87, 138.47, 137.62, 134.48, 132.29, 130.06, 127.98, 123.31, 66.03. Anal. calcd for $C_{12}H_7Br_2NO_4S$: C, 34.23; H, 1.68; N, 3.33; Found: C, 33.90; H, 1.97; N, 2.93.
**22**	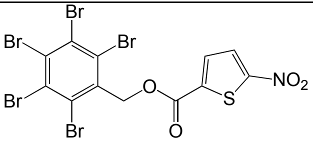	^{1}H NMR (500 MHz, $CDCl_3$) δ 7.86 ppm (d, *J* = 4.5 Hz, 1H), 7.71 (d, *J* = 4.5 Hz, 1H), 5.87 (s, 2H); ^{13}C NMR (126 MHz, $CDCl_3$) δ ppm 160.04, 155.89, 137.37, 135.78, 132.31, 131.45, 129.82, 128.80, 127.93, 71.87. Anal. calcd for $C_{12}H_4Br_5NO_4S$: C, 21.91; H, 0.61; N, 2.13; Found: C, 21.73; H, 0.90; N, 1.92.

Compound	Structure	NMR analysis
**23**	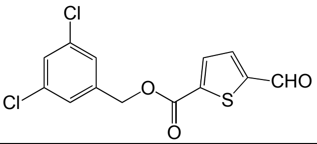	^{1}H NMR (500 MHz, $CDCl_3$) δ 9.99 ppm (s, 1H), 7.88 (d, J = 4.0 Hz, 1H), 7.75 (d, J = 4.0 Hz, 1H), 7.36 (dd, J = 2.3, 1.7 Hz, 1H), 7.31 (d, J = 1.7 Hz, 2H), 5.30 (s, 2H); ^{13}C NMR (126 MHz, $CDCl_3$) δ ppm 183.21, 161.00, 148.20, 140.15, 138.37, 135.34, 134.98, 133.88, 128.74, 126.46, 65.76. Anal. calcd for $C_{13}H_8Cl_2O_3S$: C, 49.54; H, 2.56; Found: C, 49.49; H, 2.67.
**24**	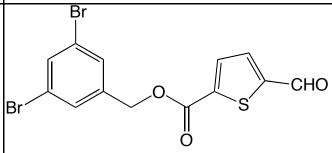	^{1}H NMR (500 MHz, $CDCl_3$) δ 9.99 ppm (s, 1H), 7.88 (d, J = 4.0 Hz, 1H), 7.75 (d, J = 4.0 Hz, 1H), 7.66 (dd, J = 1.7, 1.7 Hz, 1H), 7.51 (d, J = 1.7 Hz, 2H), 5.29 (s, 2H); ^{13}C NMR (126 MHz, $CDCl_3$) δ ppm 183.21, 160.99, 148.20, 140.13, 138.86, 134.98, 134.24, 133.89, 129.86, 123.21, 65.60. Anal. calcd for $C_{13}H_8Br_2O_3S$: C, 38.64; H, 2.00; Found: C, 38.65; H, 2.18.
**25**	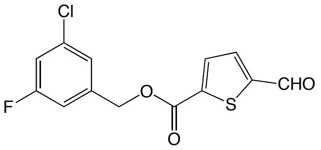	^{1}H NMR (500 MHz, $CDCl_3$) δ 9.99 ppm (s, 1H), 7.89 (d, J = 4.0 Hz, 1H), 7.75 (d, J = 4.0 Hz, 1H), 7.21 (bs, 1H), 7.09 (ddd, J = 8.5, 2.0, 2.0 Hz, 1H), 7.05 (bd, J = 8.5 Hz, 1H), 5.32 (s, 2H); ^{13}C NMR (126 MHz, $CDCl_3$) δ ppm 183.21, 162.71, 160.99, 148.19, 140.16, 138.72, 135.44, 134.98, 133.86, 123.89, 116.35, 113.42, 65.81.
**26**	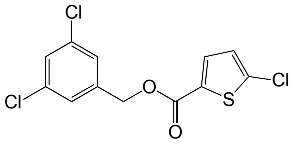	^{1}H NMR (500 MHz, $CDCl_3$) δ 7.64 ppm (d, J = 4.5 Hz, 1H), 7.34 (dd, J = 1.7, 1.7 Hz, 1H), 7.29 (d, J = 1.7 Hz, 2H), 6.96 (d, J = 3.9 Hz, 1H), 5.25 (s, 2H); ^{13}C NMR (126 MHz, $CDCl_3$) δ ppm 160.68, 138.84, 138.17, 135.29, 133.67, 131.00, 128.59, 127.46, 126.38, 65.24. Anal. calcd for $C_{12}H_7Cl_3O_2S$: C, 44.82; H, 2.19; Found: C, 44.56; H, 2.25.
**27**	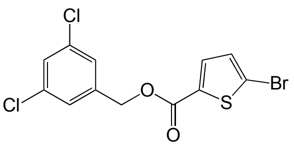	^{1}H NMR (500 MHz, $CDCl_3$) δ 7.60 ppm (d, J = 3.9 Hz, 1H), 7.34 (dd, J = 1.7, 1.7 Hz, 1H), 7.29 (d, J = 1.7 Hz, 2H), 7.10 (d, J = 4.5 Hz, 1H), 5.25 (s, 2H); ^{13}C NMR (126 MHz, $CDCl_3$) δ ppm 160.57, 138.83, 135.29, 134.32, 133.96, 131.11, 128.59, 126.37, 121.08, 65.25. Anal. calcd for $C_{12}H_7BrCl_2O_2S$: C, 39.37; H, 1.93; Found: C, 39.20; H, 2.00.
**28**	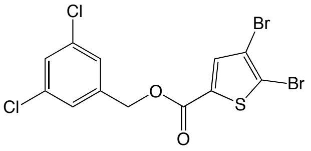	^{1}H NMR (500 MHz, $CDCl_3$) δ 7.63 ppm (s, 1H), 7.35 (dd, J = 1.7, 1.7 Hz, 1H), 7.28 (d, J = 1.7 Hz, 2H), 5.25 (s, 2H); ^{13}C NMR (126 MHz, $CDCl_3$) δ ppm 159.82, 138.43, 135.97, 135.36, 133.48, 128.76, 126.50, 119.88, 115.24, 65.62. Anal. calcd for $C_{12}H_6Br_2Cl_2O_2S$: C, 32.39; H, 1.36; Found: C, 32.23; H, 1.46.
**30**	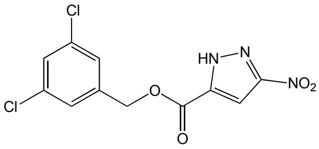	^{1}H NMR (500 MHz, $CDCl_3$) δ 11.74 ppm (s, 1H), 7.45 (s, 1H), 7.39 (dd, J = 1.7, 1.7 Hz, 1H), 7.32 (d, J = 1.7 Hz, 2H), 5.34 (s, 2H); ^{13}C NMR (126 MHz, $CDCl_3$) δ ppm, 157.45, 137.33, 135.56, 135.36, 129.25, 126.90, 105.18, 66.39. Anal. calcd for $C_{11}H_7Cl_2N_3O_4$: C, 41.80; H, 2.23; N, 13.29; Found: C, 41.72; H, 2.32; N, 13.37.
**31**	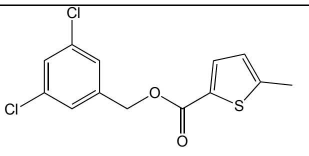	H NMR (500 MHz, $CDCl_3$) δ 7.67 ppm (d, J = 3.4 Hz, 1H), 7.32 (dd, J = 1.7, 1.7 Hz, 1H), 7.29 (d, J = 2.3 Hz, 2H), 6.79 (d, J = 3.4 Hz, 1H), 5.24 (s, 2H), 2.54 (s, 3H); ^{13}C NMR (126 MHz, $CDCl_3$) δ ppm 161.65, 148.78, 139.37, 135.17, 134.52, 130.16, 128.32, 126.56, 126.22, 64.75, 15.80. Anal. calcd for $C_{13}H_{10}Cl_2O_2S$: C, 51.84; H, 3.35; Found: C, 51.54; H, 3.32.
**33**	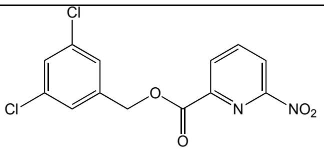	^{1}H NMR (500 MHz, $CDCl_3$) δ 8.49 ppm (d, J = 7.9 Hz, 1H), 8.46 (d, J = 8.4 Hz, 1H), 8.26 (dd, J = 7.9, 7.9 Hz, 1H), 7.38 (bs, 2H), 7.35 (dd, J = 1.7, 1.7 Hz, 1H), 5.42 (s, 2H); ^{13}C NMR (126 MHz, $CDCl_3$) δ ppm 162.88, 156.40, 147.16, 141.42, 138.26, 135.34, 130.12, 128.83, 126.82, 121.30, 66.44. Anal. calcd for $C_{13}H_8Cl_2N_2O_4$: C, 47.73; H, 2.47; N, 8.56; Found: C, 47.64; H, 2.59; N, 8.50.
**34**	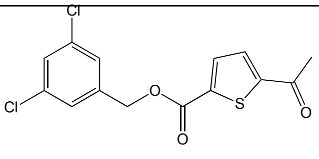	^{1}H NMR (500 MHz, $CDCl_3$) δ 7.81 ppm (d, J = 3.9 Hz, 1H), 7.65 (d, J = 3.9 Hz, 1H), 7.35 (dd, J = 2.2, 1.7 Hz, 1H), 7.31 (d, J = 1.7 Hz, 2H), 5.29 (s, 2H), 2.60 (s, 3H); ^{13}C NMR (126 MHz, $CDCl_3$) δ ppm 190.72, 161.15, 149.30, 138.82, 138.55, 135.33, 134.01, 131.69, 128.69, 126.44, 65.61, 27.04. Anal. calcd for $C_{14}H_{10}Cl_2O_3S$: C, 51.08; H, 3.06; Found: C, 50.71; H, 3.10.
**36**	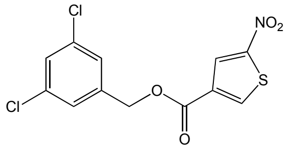	^{1}H NMR (500 MHz, $CDCl_3$) δ 8.30 ppm (d, J = 1.7 Hz, 1H), 8.29 (d, J = 1.7 Hz, 1H), 7.36 (dd, J = 1.7, 1.7 Hz, 1H), 7.30 (d, J = 1.7 Hz, 2H), 5.28 (s, 2H); ^{13}C NMR (126 MHz, $CDCl_3$) δ ppm 160.47, 152.39, 138.38, 137.60, 135.40, 132.13, 128.86, 128.22, 126.6, 65.66. Anal. calcd for $C_{12}H_7Cl_2NO_4S$: C, 43.39; H, 2.12; N, 4.22; Found: C, 43.18; H, 2.29; N, 4.10.

Compound	Structure	NMR analysis
**37**	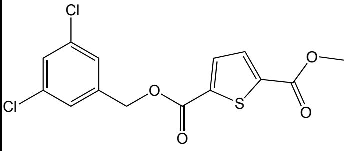	^{1}H NMR (500 MHz, $CDCl_3$) δ 7.78 ppm (d, *J* = 4.0 Hz, 1H), 7.75 (d, *J* = 4.0 Hz, 1H), 7.35 (dd, *J* = 1.7, 1.7 Hz, 1H), 7.31 (d, *J* = 1.7 Hz, 2H), 5.28 (s, 2H), 3.92 (s, 3H); ^{13}C NMR (126 MHz, $CDCl_3$) δ ppm 161.90, 161.08, 139.39, 138.60, 138.02, 135.33, 133.65, 133.15, 128.68, 126.45, 65.55, 52.66.
**39**	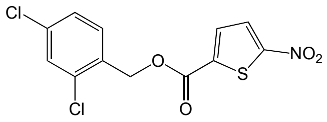	^{1}H NMR (500 MHz, $CDCl_3$) δ 7.87 ppm (d, *J* = 4.5 Hz, 1H), 7.73 (d, *J* = 4.5 Hz, 1H), 7.46 (d, *J* = 1.7 Hz, 1H), 7.42 (d, *J* = 8.4 Hz, 1H), 7.30 (dd, *J* = 8.1, 2.0 Hz, 1H), 5.43 (s, 2H); ^{13}C NMR (126 MHz, $CDCl_3$) δ ppm 160.23, 155.75, 137.84, 135.55, 134.87, 132.14, 131.23, 131.10, 129.76, 127.97, 127.44, 64.67. Anal. calcd for $C_{12}H_7Cl_2NO_4S$: C, 43.39; H, 2.12; N, 4.22; Found: C, 43.27; H, 2.23; N, 4.11.
**40**	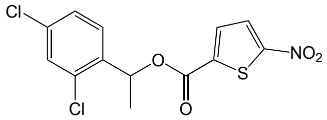	^{1}H NMR (500 MHz, $CDCl_3$) δ 7.88 ppm (d, *J* = 4.5 Hz, 1H), 7.73 (d, *J* = 4.5 Hz, 1H), 7.43 (d, *J* = 8.5 Hz, 1H), 7.42 (s, 1H), 7.30 (dd, *J* = 8.5, 1.7 Hz, 1H), 6.38 (q, *J* = 6.8 Hz, 1H), 1.66 (d, *J* = 6.8 Hz, 3H); ^{13}C NMR (126 MHz, $CDCl_3$) δ ppm 159.52, 155.60, 138.17, 137.04, 134.62, 132.83, 132.01, 129.70, 128.00, 127.77, 127.55, 71.22, 20.92. Anal. calcd for $C_{13}H_9Cl_2NO_4S$: C, 45.10; H, 2.62; N, 4.05; Found: C, 44.94; H, 2.63; N, 3.91.
**41**	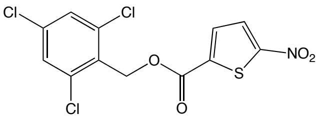	^{1}H NMR (500 MHz, $CDCl_3$) δ 7.86 ppm (d, *J* = 4.5 Hz, 1H), 7.70 (d, *J* = 4.5 Hz, 1H), 7.42 (s, 2H), 5.60 (s, 2H); ^{13}C NMR (126 MHz, $CDCl_3$) δ ppm 160.19, 155.73, 137.71, 137.57, 136.29, 132.13, 129.07, 128.68, 127.93, 62.21.
**42**	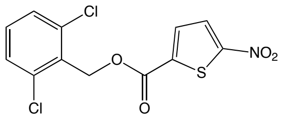	^{1}H NMR (500 MHz, $CDCl_3$) δ 7.85 ppm (d, *J* = 3.9 Hz, 1H), 7.70 (d, *J* = 3.9 Hz, 1H), 7.39 (d, *J* = 8.4 Hz, 2H), 7.30 (dd, *J* = 8.4, 7.3 Hz, 1H), 5.66 (s, 2H); ^{13}C NMR (126 MHz, $CDCl_3$) δ ppm 160.30, 155.65, 138.01, 137.09, 132.03, 131.06, 130.36, 128.62, 127.91, 62.82.
**43**	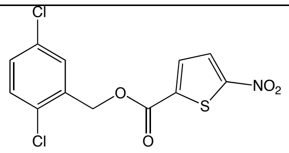	^{1}H NMR (500 MHz, $CDCl_3$) δ 7.89 ppm (d, *J* = 4.5 Hz, 1H), 7.75 (d, *J* = 4.5 Hz, 1H), 7.46 (d, *J* = 2.2 Hz, 1H), 7.37 (d, *J* = 8.4 Hz, 1H), 7.31 (dd, *J* = 8.4, 2.2 Hz, 1H), 5.43 (s, 2H); ^{13}C NMR (126 MHz, $CDCl_3$) δ ppm 160.16, 155.82, 137.70, 134.16, 133.02, 132.24, 132.07, 130.94, 130.10, 129.94, 127.99, 64.60.
**44**	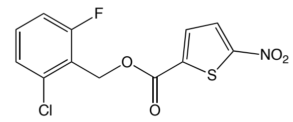	^{1}H NMR (500 MHz, $CDCl_3$) δ 7.85 ppm (d, *J* = 3.9 Hz, 1H), 7.70 (d, *J* = 4.5 Hz, 1H), 7.38 – 7.33 (m, 1H), 7.29 – 7.26 (m, 1H), 7.11 – 7.05 (m, 1H), 5.54 (d, *J* = 1.7 Hz, 2H); ^{13}C NMR (126 MHz, $CDCl_3$) δ ppm 162.03, 160.27, 155.65, 138.03, 136.54, 132.02, 131.40, 127.90, 125.68, 120.68, 114.48, 58.85.
**45**	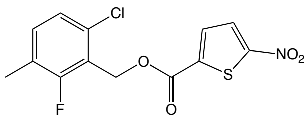	^{1}H NMR (500 MHz, $CDCl_3$) δ 7.85 ppm (d, *J* = 4.0 Hz, 1H), 7.69 (d, *J* = 4.0 Hz, 1H), 7.28 (dd, *J* = 8.5, 6.2 Hz, 1H), 6.99 (dd, *J* = 8.5, 8.5 Hz, 1H), 5.55 (d, *J* = 1.7 Hz, 2H), 2.39 (s, 3H); ^{13}C NMR (126 MHz, $CDCl_3$) δ ppm 160.32, 160.16, 155.59, 138.17, 136.21, 132.88, 132.36, 131.96, 127.90, 120.32, 113.80, 59.37, 20.10.
**46**	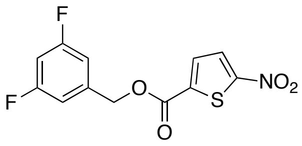	^{1}H NMR (500 MHz, $CDCl_3$) δ 7.89 ppm (d, *J* = 4.5 Hz, 1H), 7.75 (d, *J* = 4.5 Hz, 1H), 6.98 – 6.91 (m, 2H), 6.85 – 6.78 (m, 1H), 5.34 (s, 2H); ^{13}C NMR (126 MHz, $CDCl_3$) δ ppm 164.19, 162.16, 160.21, 155.84, 138.45, 137.66, 132.25, 127.99, 111.00, 110.84, 104.20, 66.33.
**47**	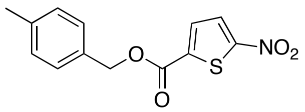	^{1}H NMR (500 MHz, $CDCl_3$) δ 7.85 ppm (d, *J* = 3.9 Hz, 1H), 7.69 (d, *J* = 3.9 Hz, 1H), 7.32 (d, *J* = 7.9 Hz, 2H), 7.21 (d, *J* = 7.9 Hz, 2H), 5.33 (s, 2H), 2.37 (s, 3H); ^{13}C NMR (126 MHz, $CDCl_3$) δ ppm 160.53, 155.48, 138.80, 138.64, 131.76, 129.45, 128.67, 127.93, 68.00, 21.25.

Compound	Structure	NMR analysis
**48**	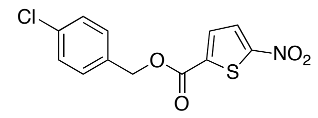	^{1}H NMR (500 MHz, $CDCl_3$) δ 7.86 ppm (d, J = 3.9 Hz, 1H), 7.71 (d, J = 3.9 Hz, 1H), 7.37 (s, 4H), 5.33 (s, 2H); ^{13}C NMR (126 MHz, $CDCl_3$) δ ppm 160.39, 155.64, 138.13, 134.85, 133.22, 131.99, 129.91, 129.03, 127.95, 67.13.
**49**	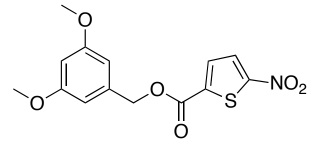	^{1}H NMR (500 MHz, $CDCl_3$) δ 7.86 ppm (d, J = 4.5 Hz, 1H), 7.72 (d, J = 4.5 Hz, 1H), 6.55 (d, J = 2.2 Hz, 2H), 6.45 (dd, J = 2.2, 2.2 Hz, 1H), 5.30 (s, 2H), 3.81 (s, 6H); ^{13}C NMR (126 MHz, $CDCl_3$) δ ppm 161.09, 160.43, 155.58, 138.40, 136.92, 131.90, 127.96, 106.25, 100.44, 67.82, 55.43.
**50**	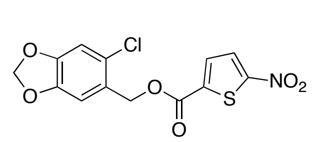	^{1}H NMR (500 MHz, $CDCl_3$) δ 7.86 ppm (d, J = 3.9 Hz, 1H), 7.71 (d, J = 3.9 Hz, 1H), 6.94 (s, 1H), 6.90 (s, 1H), 6.01 (s, 2H), 5.37 (s, 2H); ^{13}C NMR (126 MHz, $CDCl_3$) δ ppm 160.38, 155.62, 148.82, 146.87, 138.22, 131.95, 127.95, 126.76, 125.36, 110.27, 110.22, 102.13, 65.42.
**51**	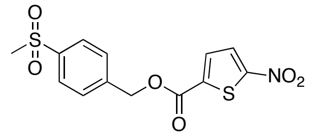	^{1}H NMR (500 MHz, $CDCl_3$) δ 8.00 ppm (d, J = 7.9 Hz, 2H), 7.89 (d, J = 4.5 Hz, 1H), 7.75 (d, J = 4.5 Hz, 1H), 7.63 (d, J = 7.9 Hz, 2H), 5.46 (s, 2H), 3.07 (s, 3H); ^{13}C NMR (126 MHz, $CDCl_3$) δ ppm 160.25, 155.86, 155.19, 140.90, 140.86, 137.53, 132.31, 128.85, 127.99, 66.57, 44.49.
**52**	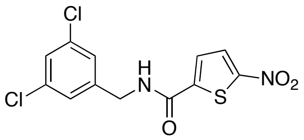	^{1}H NMR (500 MHz, $CDCl_3$) δ 7.87 ppm (d, J = 3.9 Hz, 1H), 7.40 (d, J = 3.9 Hz, 1H), 7.32 (dd, J = 1.7, 2.2 Hz, 1H), 7.23 (d, J = 1.7 Hz, 2H), 6.44 (bs, 1H), 4.59 (d, J = 5.6 Hz, 2H); ^{13}C NMR (126 MHz, $CDCl_3$) δ ppm 160.02, 154.12, 145.84 , 143.70, 140.46, 135.53, 128.26, 128.08, 126.34, 43.30.
**53**	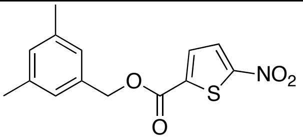	^{1}H NMR (500 MHz, $CDCl_3$) δ 7.85 ppm (d, J = 4.5 Hz, 1H), 7.71 (d, J = 4.5 Hz, 1H), 7.03 (bs, 2H), 7.01 (bs, 1H), 5.29 (s, 2H), 2.34 (s, 6H); ^{13}C NMR (126 MHz, $CDCl_3$) δ ppm 160.54, 155.49, 138.65, 138.44, 134.60, 131.79, 130.46, 127.94, 126.34, 68.12, 21.25.
**54**	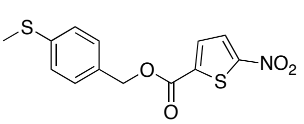	^{1}H NMR (500 MHz, $CDCl_3$) δ 7.85 ppm (d, J = 3.9 Hz, 1H), 7.70 (d, J = 3.9 Hz, 1H), 7.35 (d, J = 7.9 Hz, 2H), 7.27 (d, J = 7.9 Hz, 2H), 5.32 (s, 2H), 2.49 (s, 3H); ^{13}C NMR (126 MHz, $CDCl_3$) δ ppm 160.49, 155.53, 139.75, 138.45, 131.85, 131.30, 129.22, 127.94, 126.52, 67.68, 15.59.
**55**	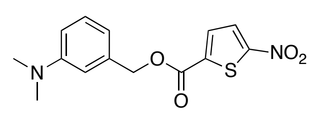	^{1}H NMR (500 MHz, $CDCl_3$) δ 7.85 ppm (d, J = 4.0 Hz, 1H), 7.70 (d, J = 4.0 Hz, 1H), 7.26 (dd, J = 7.9, 7.4 Hz, 1H), 6.77 (m, 3H), 5.32 (s, 2H), 2.98 (s, 6H); ^{13}C NMR (126 MHz, $CDCl_3$) δ ppm 160.53, 155.43, 150.69, 138.70, 135.55, 131.73, 129.49, 127.92, 116.56, 112.84, 112.33, 68.59, 40.54.
**56**	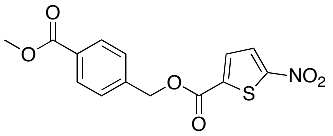	^{1}H NMR (500 MHz, $CDCl_3$) δ 8.07 ppm (d, J = 8.4 Hz, 2H), 7.87 (d, J = 4.5 Hz, 1H), 7.73 (d, J = 4.5 Hz, 1H), 7.49 (d, J = 8.4 Hz, 2H), 5.42 (s, 2H), 3.93 (s, 3H); ^{13}C NMR (126 MHz, $CDCl_3$) δ ppm 166.53, 160.31, 155.69, 139.59, 137.94, 132.05, 130.48, 130.03, 127.93, 67.08, 52.23.
**57**	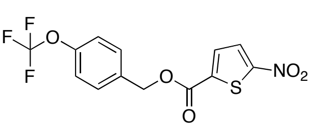	^{1}H NMR (500 MHz, $CDCl_3$) δ 7.87 ppm (d, J = 4.5 Hz, 1H), 7.72 (d, J = 4.5 Hz, 1H), 7.47 (d, J = 7.9 Hz, 2H), 7.25 (d, J = 7.9 Hz, 2H), 5.37 (s, 2H); ^{13}C NMR (126 MHz, $CDCl_3$) δ ppm 160.37, 155.67, 149.48, 138.04, 133.42, 132.02, 130.06, 127.93, 121.23, 120.40 , 66.93.
